# Supplementary material for: GWAS in childhood acute lymphoblastic leukemia reveals novel genetic associations at chromosomes 17q12 and 8q24.21
Source: Nat Commun. 2018 Jan 18;9:286. doi: 10.1038/s41467-017-02596-9 (PMC5773513; doi:10.1038/s41467-017-02596-9)
Supplement: Supplementary file 1 — Supplementary Information [file 41467_2017_2596_MOESM1_ESM.pdf]

Supplementary Table 1: Sample counts for GWAS analysis

Discovery	Latino	Non-Latino white	African American	total
Cases CCRLP	1949	1184	130	3263
Controls				
CCRLP	2120	1241	145	15977
Kaiser	6464	2310	3697	
Replication				
Cases CCLS	530	0	0	530
COG	0	959	0	959
Controls CCLS	511	0	0	511
WTCCC	0	2624	0	2624

CCRLP: California Childhood Cancer Records Linkage Project
Kaiser: Kaiser Genetic Epidemiology Research on Aging
CCLS: California Childhood Leukemia Study
COG: Children's Oncology Group
WTCCC: Welcome Trust Case Control Consortium

Supplementary Table 2: Case-control comparisons at the known "top hit" childhood ALL loci from prior publications

[a] Risk allele frequency (RAF) from 1000 Genomes (Amerindian RAF is shown for Latinos)
[b] Sample sizes for all analyses: CCRLP Latinos: 1949 cases, 8584 controls; CCRLP whites: 1184 cases, 3551 controls; CCRLP African Americans: 130 cases, 3842 controls.

chromosome

7

	**IKZF1- rs11978267 risk allele A/G**		
	**RAF[a]**	**P-value**	**OR (95% CI)**
CCRLP Latinos [b]	0.77	$9.1x10^{-10}$	1.29 (1.19-1.40)
CCRLP Whites[b]	0.68	$8.9x10^{-23}$	1.66 (1.50-1.84)
CCRLP African-Americans[b]	0.83	$5.5x10^{-3}$	1.49 (1.12-1.98)
**CCRLP Meta-analysis**		$2.2x10^{-29}$	1.43

8

	**GATA3 - rs3824662 risk allele G/T**		
	**RAF[a]**	**P-value**	**OR (95% CI)**
CCRLP Latinos	0.92	0.84	1.03 (0.76-1.4)
CCRLP Whites	0.81	0.07	1.34 (0.97-1.85)
CCRLP African-Americans	0.64	0.04	1.84 (1.02-3.32)
**CCRLP Meta-analysis**		0.045	1.24

9

	**CDKN2A- rs2069426 risk allele A/C**		
	**RAF[a]**	**P-value**	**OR (95% CI)**
CCRLP Latinos	0.045	$1.1x10^{-3}$	1.28 (1.10-1.48)
CCRLP Whites	0.081	$6.5x10^{-7}$	1.47 (1.26-1.70)
CCRLP African-Americans	0.099	$5.5x10^{-3}$	1.27 (0.83-1.92)
**CCRLP Meta-analysis**		$3.7x10^{-9}$	1.35

10

	PIP4K2A- rs4748813 risk allele C/T		
	**RAF[a]**	**P-value**	**OR (95% CI)**
CCRLP Latinos	0.76	$9.8x10^{-11}$	0.74 (0.68-0.81)
CCRLP Whites	0.61	$4.1x10^{-9}$	0.75 (0.68-0.82)
CCRLP African-Americans	0.35	$2.0x10^{-3}$	0.67 (0.51-0.86)
**CCRLP Meta-analysis**		$2.5x10^{-20}$	0.74

10

	ARID5B - rs7089424 risk allele T/G		
	**RAF[a]**	**P-value**	**OR (95% CI)**
CCRLP Latinos	0.48	$3.6x10^{-22}$	1.71 (1.58-1.84
CCRLP Whites	0.33	$1.0x10^{-43}$	1.60 (1.46-1.77)
CCRLP African-Americans	0.24	0.51	1.10 (0.83-1.47)
**CCRLP Meta-analysis**		$2.20x10^{-62}$	1.64

14

	CEBPE- rs2239633 risk allele C/T		
	**RAF[a]**	**P-value**	**OR (95% CI)**
CCRLP Latinos	0.55	$5.6x10^{-10}$	0.78 (0.72-0.84)
CCRLP Whites	0.52	$2.0x10^{-4}$	0.80 (0.72-0.90)
CCRLP African-Americans	0.82	0.58	0.92 (0.67-1.25)
**CCRLP Meta-analysis**		$7.3x10^{-13}$	0.79

The following two loci reported in Vijayakrishnan, et al., Leukemia 2017

10

	LHPP- *rs3740540 risk allele C/T		
	**RAF[a]**	**P-value**	**OR (95% CI)**
CCRLP Latinos	0.75	$1.7x10^{-4}$	1.16 (1.07-1.25)
CCRLP Whites	0.7	$1.2x10^{-2}$	1.17 (1.04-1.32)
CCRLP African-Americans	0.95	0.54	1.09 (0.83-1.43)
**CCRLP Meta-analysis**		$5.7x10^{-6}$	1.16

*imputed genotype

12

	ELK3- *rs2302902 risk allele A/G		
	**RAF[a]**	**P-value**	**OR (95% CI)**
CCRLP Latinos	0.34	1.7x10-3	1.13 (1.05-1.22)
CCRLP Whites	0.23	0.22	1.07 (0.96-1.18)
CCRLP African-Americans	0.22	0.69	0.95 (0.73-1.23)
**CCRLP Meta-analysis**		$2.1x10^{-3}$	1.1

*imputed genotype

Supplementary Table 3: Lead SNPs near SP4, 8q24, and IKZF3 reaching genome-wide significance in discovery analyses of CCRLP acute lymphoblastic leukemia patients, including replication analyses in the Children's Oncology Group and the California Childhood Leukemia Study Latino Participants: P values, Odds Ratios, and I^2 values.

	**SP4 - rs2390536 risk allele A**				**8q24.1 - rs4617118 risk allele G**				**IKZF3 - rs2290400 risk allele T**		
	**P-value**	**OR (95% CI)**	**I^2**		**P-value**	**OR (95% CI)**	**I^2**		**P-value**	**OR (95% CI)**	**I^2**
**CCRLP Meta-analysis**	**3.59×10^{-8}**	1.20 (1.13-1.29)	0%		**3.05×10^{-9}**	1.27 (1.17-1.38)	7.2%		**2.05×10^{-8}**	1.18 (1.11-1.25)	0%
CCRLP Hispanics	1.42×10^{-4}	1.19 (1.09-1.31)	-		2.90×10^{-5}	1.25 (1.12-1.38)	-		4.33×10^{-6}	1.20 (1.11-1.30)	-
CCRLP Whites	5.69×10^{-5}	1.23 (1.11-1.35)	-		1.74×10^{-3}	1.26 (1.09-1.45)	-		2.72×10^{-3}	1.15 (1.05-1.26)	-
CCRLP African-Americans	0.73	1.07 (0.73-1.58)	-		1.55×10^{-3}	1.54 (1.18-2.01)	-		0.18	1.19 (0.92-1.53)	-
**Replication meta-analysis**	0.075	1.10 (0.99-1.23)	48.3%		**1.29×10^{-4}**	1.30 (1.14-1.49)	60.8%		**0.013**	1.14 (1.03-1.26)	0%
COG Replication (European)	0.025	1.15 (1.018-1.31)	-		0.011	1.22 (1.05-1.43)	-		0.13	1.10 (0.97-1.25)	-
CCLS Replication (Hispanic)	0.74	0.96 (0.77-1.20)	-		1.03×10^{-3}	1.58 (1.20-2.07)	-		0.030	1.22 (1.02-1.46)	-
**Combined Datasets**	1.77×10^{-8}	1.18 (1.11-1.24)	5.8%		1.76×10^{-12}	1.28 (1.19-1.37)	16.4%		1.06×10^{-9}	1.17 (1.11-1.23)	0%

See Table 1 of main text for information regarding whether SNPs are genotyped “on array” or imputed for each sample set.

Supplementary Table 4: Top hit SNPs (1 X 10^{-7} or lower) proximal to the top hit SNP rs4617118 in chromosome 8 with functional annotations.

rsID	pos	allele_A	allele_B	P_value	coded_af	beta	OR	AFR	AMR	ASN	EUR	DNAse	Regulome DB*	gwas	Motifs	RefSeq	Location
rs72730213	130154246	G	A	4.43E-09	0.136035	0.252892	1.28774419	0.01	0.09	0.04	0.13		7	.			
rs72730214	130155389	T	C	1.54E-08	0.151237	0.234029	1.26368114	0.03	0.11	0.09	0.15		7	.			
rs11777951	130155422	A	G	4.34E-09	0.136086	0.252682	1.2874738	0.01	0.09	0.04	0.13		7	.			
rs72730215	130155860	C	T	4.30E-09	0.136107	0.25262	1.28739397	0.01	0.09	0.04	0.13		5	.	Hgf		
**rs4617118**	**130156143**	**A**	**G**	**3.05E-09**	**0.185412**	**0.229802**	**1.25835083**	**0.28**	**0.13**	**0.09**	**0.15**		6	**.**	**Mef2**		
rs72730217	130156776	A	C	1.44E-08	0.158423	0.231295	1.26023095	0.12	0.11	0.09	0.15		7	.			
rs4418312	130157804	C	G	1.75E-09	0.153064	0.246928	1.28008694	0.06	0.1	0.09	0.15		7	.			
rs72730218	130162156	A	G	1.25E-09	0.150696	0.251841	1.28639149	0.06	0.11	0.07	0.15		7	.			
rs149184189	130169385	C	G	2.19E-08	0.13945	0.244254	1.27666856	0.01	0.1	0.06	0.14		7	.			
rs11784062	130169962	T	G	3.39E-08	0.131708	0.248368	1.2819316	0.02	0.1	0.04	0.13		6	.	SP4, WT1, Zfp281, Sp1, MAZ, CKROX, MAZR, A549, Sp3, UF1H3BETA, KLF16, Plagl1		
rs77269791	130176904	G	A	1.61E-09	0.0947452	0.311346	1.36526152	0.01	0.06	0.0035	0.1	Yes	4	.	T47d, Rwpe1, Htr8, Ut189, POLR2A		
rs11994022	130178201	C	T	8.79E-08	0.147707	0.228485	1.25669468	0.02	0.1	0.06	0.14	Yes	5	.	Hoxb9, Hoxa9, MEF2A, Hoxc12, MEF-2, Cdx-2, hoxa9, Fibrop		
rs79207438	130182671	C	T	2.29E-08	0.115747	0.257541	1.29374485	0.05	0.08	0.02	0.11	Yes	3a	.	Bbx, Lhcnm2, Diff4d, Lhcnm2, Htr8, SETDB1		
rs74756667	130182794	G	A	3.65E-08	0.118482	0.2517	1.28621012	0.05	0.09	0.02	0.11		5	.	Hbvp, Htr8		
rs75777619	130185176	A	G	2.26E-08	0.115729	0.257615	1.29384059	0.05	0.08	0.02	0.11	Yes	5	.	PPARalpha:RXRalpha		
rs28665337	130194104	C	A	1.74E-08	0.116628	0.257823	1.29410974	0.05	0.09	0.02	0.11		7	.			
rs28484683	130194436	G	A	2.71E-08	0.119517	0.252192	1.28684309	0.07	0.1	0.02	0.11		7	.			
rs76999656	130194640	G	C	2.64E-08	0.119396	0.252637	1.28741586	0.07	0.1	0.02	0.11		6	.	HNF4;NF-kappaB		
rs76714314	130195170	G	A	4.80E-08	0.0954829	0.276596	1.31863354	0.01	0.06	0	0.1		7	.			
rs77387362	130195769	T	C	2.73E-08	0.11952	0.252104	1.28672985	0.07	0.1	0.02	0.11		6	.	Evi-1;Foxa;Foxa;Foxa;Foxj2;Foxl1;Foxp1;HDAC2;Mef2;Sox_3		
rs75614243	130197102	A	G	2.75E-08	0.119523	0.252023	1.28662563	0.07	0.1	0.02	0.11		6	.	Bbx;NF-Y;RXRA		
rs11997664	130197527	C	T	2.76E-08	0.119523	0.25199	1.28658317	0.07	0.1	0.02	0.11		6	.	THAP1;YY1		
rs6995223	130200252	G	C	2.82E-08	0.119528	0.251749	1.28627314	0.07	0.1	0.02	0.11		7	.			
rs7830899	130201569	C	T	3.09E-08	0.119586	0.250998	1.28530751	0.07	0.1	0.02	0.11		5	.	Foxp1;Hbp1;Irf;Nrf-2;Pax-6;Zfp105;p300		
rs6470703	130201966	T	C	2.41E-09	0.123666	0.268962	1.30860541	0.11	0.1	0.02	0.11		7	.			
rs3935421	130203139	A	G	2.44E-09	0.123667	0.268836	1.30844054	0.11	0.1	0.02	0.11		6	.	ATF3;ATF3;ATF3;ATF6;Hdx;XBP-1		
rs73388076	130204575	C	G	2.50E-09	0.123669	0.268597	1.30812786	0.11	0.1	0.02	0.11	Yes	5	.	Nkx2;Rhox11		
rs73388080	130204909	C	T	4.23E-09	0.124452	0.264184	1.30236781	0.11	0.1	0.02	0.11		7	.			
rs7460736	130205817	A	T	2.62E-09	0.123672	0.268057	1.30742166	0.11	0.1	0.02	0.11		5	.	Dbx1;Fox;Foxa;Foxa;Foxc1;Foxf1;Foxi1;Foxj1;Foxk1;Foxl1;Foxo3;Foxp1;HDAC2;Hoxd8;Ncx;Zfp105		
rs4557668	130206261	G	C	3.58E-08	0.11967	0.249486	1.2833656	0.07	0.1	0.02	0.11		7	.			
rs78165253	130207410	A	G	5.58E-08	0.119482	0.245779	1.27861697	0.07	0.1	0.02	0.11		7	.			
rs80029873	130207750	G	A	5.61E-08	0.119483	0.245722	1.27854409	0.07	0.1	0.02	0.11	Yes	5	.	ATF3;Nrf-2;SREBP;ZEB1;Zbtb3		
rs78110386	130209209	C	A	4.01E-08	0.118945	0.248735	1.28240215	0.07	0.1	0.02	0.11	Yes	5	.	CTCF;HNF4		
rs11994734	130209964	T	C	3.66E-08	0.116626	0.251139	1.28548876	0.04	0.09	0.02	0.11		7	.			
rs55806657	130210430	T	C	4.71E-09	0.123597	0.263355	1.30128859	0.11	0.1	0.02	0.11		5	.	BATF;Bach1;TCF11;MafG;Zbtb3		
rs4571701	130210946	G	C	5.87E-08	0.119488	0.245214	1.27789475	0.07	0.1	0.02	0.11		7	.			
rs4422741	130211328	T	C	5.91E-08	0.119488	0.245139	1.27779892	0.07	0.1	0.02	0.11		3a	.	CEBPG;p300		
rs75114317	130214963	C	A	3.95E-08	0.116632	0.25031	1.28442353	0.04	0.09	0.02	0.11	Yes	4	.	CDP;Cphx;Pbx-1		
rs56397715	130217355	A	G	4.07E-08	0.116635	0.249934	1.28394067	0.04	0.09	0.02	0.11	Yes	6	.	Dbx1;HNF1;HNF1;HNF1;Hoxb4;Pou6f1		
rs75281442	130218345	T	C	4.14E-08	0.116636	0.249764	1.28372242	0.04	0.09	0.02	0.11	Yes	3a	.	Cphx		
rs11997752	130218781	T	C	4.17E-08	0.116637	0.249677	1.28361074	0.04	0.09	0.02	0.11	Yes	6	.	Cdx;Evi-1;Foxj1;Foxj2;Foxk1;Foxp1;HLF		
rs11994768	130219021	G	A	6.57E-08	0.119501	0.243903	1.27622053	0.07	0.1	0.02	0.11		7	.			
rs5003704	130222435	G	A	4.26E-08	0.116622	0.249584	1.28349137	0.04	0.09	0.02	0.11	Yes	7	.			
rs4484652	130223575	G	T	5.45E-08	0.119086	0.247633	1.28098972	0.07	0.1	0.02	0.11		7	.			
rs59047450	130224231	A	G	1.89E-08	0.135731	0.245163	1.27782958	0.12	0.1	0.05	0.13		5	.	Irf;PU.1;Rad21		
rs59282112	130225521	T	A	5.06E-08	0.136049	0.238553	1.26941098	0.08	0.1	0.05	0.14		7	.			
rs7832380	130226561	G	A	8.19E-08	0.128426	0.248125	1.28162013	0.08	0.09	0.05	0.12		7	.			

SNPs having a larger effect size than the index SNP with the lowest P-value rs4617118 (bolded) appear here. Position (hg19) followed by the major allele A, minor allele B, P-value in meta-analysis,
coded allele frequency in entire dataset, beta value, meta-analysis OR (odds ratio), 1000 genomes minor allele frequency in Africans, Amerindians, Asians, and Europeans,
evidence of DNA hypersensitive site (from Haploreg), Regulome DB score, GWAS hits from other diseases, ENCODE-described motifs which interact at the site, RefSeq gene annotation at that location, and the Location within the gene.

*Regulome DB Score

1a	eQTL + TF binding + matched TF motif + matched DNase Footprint + DNase peak
1b	eQTL + TF binding + any motif + DNase Footprint + DNase peak
1c	eQTL + TF binding + matched TF motif + DNase peak
1d	eQTL + TF binding + any motif + DNase peak
1e	eQTL + TF binding + matched TF motif
1f	eQTL + TF binding / DNase peak
2a	TF binding + matched TF motif + matched DNase Footprint + DNase peak
2b	TF binding + any motif + DNase Footprint + DNase peak
2c	TF binding + matched TF motif + DNase peak
3a	TF binding + any motif + DNase peak
3b	TF binding + matched TF motif
4	TF binding + DNase peak
5	TF binding or DNase peak
6	other (minimal binding data)
7	no data

Supplementary Table 5: Top hit SNPs (1 X 10^{-7} or lower) proximal to the top hit SNP rs2290400 in chromosome 17, with functional annotations.

rsID	pos	allele_A	allele_B	P_value	coded_af	beta	OR	chr	AFR	AMR	ASN	EUR	DNAse	regulome DB	gwas	Motifs Altered	RefSeq	Location
rs2941519	37903731	G	A	7.35E-07	0.547115	0.145409	1.15651249	17	0.33	0.57	0.62	0.49		7			GRB7	
rs9747973	37905107	C	T	5.08E-07	0.53923	0.14552	1.15664087	17	0.41	0.56	0.63	0.47	Yes	5		TBX21, T47d	GRB7	
rs2941522	37910368	C	T	6.67E-07	0.545668	0.143793	1.15464507	17	0.44	0.58	0.65	0.47	Yes	4		NRSF	GRB7	
rs907091	37921742	C	T	4.92E-08	0.533186	0.160323	1.17388998	17	0.31	0.56	0.65	0.48	Yes	1f		MED24, SPI1, GATA2	IKZF3	U3
rs2952140	37928059	C	T	9.56E-08	0.530094	0.156238	1.16910442	17	0.32	0.56	0.64	0.47		5		ATF3;GATA;Pax-5;SREBP	IKZF3	INT
rs2313430	37929816	T	C	2.49E-07	0.535753	0.150379	1.16227466	17	0.38	0.56	0.65	0.47	Yes	1d		Pou2f2	IKZF3	INT
rs2952144	37960017	C	T	6.30E-07	0.533532	0.145907	1.15708857	17	0.38	0.55	0.65	0.47		6		Myc;NRSF;PLAG1;Sp4	IKZF3	INT
rs9303277	37976469	C	T	1.39E-07	0.463669	-0.154423	0.85690947	17	0.62	0.44	0.35	0.53	Yes	1f	Biliary cirrhosis, Lupus	Arid3a;Cdc5;Pou2f2;Pou3f1	IKZF3	INT
rs12944882	37983492	T	C	1.38E-07	0.482766	-0.153458	0.85773679	17	0.64	0.46	0.32	0.56		7			IKZF3	INT
rs3816470	37985801	A	G	1.58E-07	0.480899	-0.153492	0.85770763	17	0.64	0.45	0.32	0.56		6		Dbx1;Fox;Foxl1;Foxo3;HNF1;Pou2f2;TATA;TEF	IKZF3	INT
rs9916765	38005595	T	C	2.40E-07	0.451725	-0.152755	0.85833999	17	0.62	0.44	0.31	0.52		6		AP-1;IRC900814;Pax-4;Pax-4	IKZF3	INT
rs35222145	38007321	T	G	1.88E-07	0.35437	-0.182204	0.8334313	17	0.32	0.35	0.25	0.44		6		Mef2;Pax1;Pou2f2;TATA	IKZF3	INT
rs1453559	38020419	T	C	8.93E-07	0.461295	-0.14168	0.86789894	17	0.63	0.44	0.31	0.53	Yes	4		SRF; Htr8; Hcf	IKZF3	U5
rs11655198	38026169	C	T	4.48E-07	0.416013	-0.148681	0.861844	17	0.26	0.39	0.31	0.52		6		Gmeb1	ZPBP2	INT
rs11650661	38026286	A	T	6.46E-07	0.418145	-0.145891	0.8642519	17	0.26	0.39	0.31	0.52		7			ZPBP2	INT
rs11655292	38026361	C	G	6.65E-07	0.416905	-0.146054	0.86411104	17	0.26	0.39	0.31	0.52		7			ZPBP2	INT
rs12936231	38029120	C	G	3.08E-07	0.455691	-0.149981	0.86072433	17	0.56	0.43	0.31	0.53	Yes	1f		Foxm1;Lmo2;ZEB1;Zbtb3	ZPBP2	INT
rs9903250	38031030	G	A	5.19E-07	0.418388	-0.14758	0.86279341	17	0.27	0.4	0.31	0.52		7			ZPBP2	INT
rs20021613[illegible]	38032132	G	GC	8.56E-08	0.423408	-0.159103	0.8529085	17	0.26	0.4	0.31	0.52		6		HDAC2;Irf;Pax-4;SP1;Zfp105;p300	ZPBP2	INT
rs9901146	38043343	G	A	9.77E-07	0.428794	-0.144359	0.86557695	17	0.37	0.4	0.31	0.52	Yes	6		CDP_2; ELF3	ZPBP2	.
rs36084703	38063980	CA	C	7.25E-07	0.384373	-0.149846	0.86084054	17	0.13	0.37	0.3	0.49		6		Bbx;Dbx2;Evi-1;HMG-IY;Hoxd10;Lhx3;Lhx3;Mef2;Ncx2;Nkx6;Pax4;Sox_18	GSDMB	INT
**rs2290400**	**38066240**	**T**	**C**	**1.29E-08**	**0.44566**	**-0.166431**	**0.84668124**	**17**	**0.49**	**0.42**	**0.33**	**0.52**		**6**	**Diabetes, type 1; asthma**	**PRDM1**	**GSDMB**	**INT**
rs56380902	38066372	T	C	4.38E-07	0.440646	-0.145633	0.86447491	17	0.47	0.42	0.32	0.52	Yes	7			GSDMB	INT
rs7219923	38074518	C	T	1.33E-07	0.551585	0.154014	1.16650722	17	0.53	0.58	0.68	0.47	Yes	1f		MED24; Ifna4h; POLR2A	GSDMB	INT

SNPs having a larger effect size than the index SNP with the lowest P-value rs2290400 (bolded) appear here. Position (hg19) followed by the major allele A, minor allele B, P-value in meta-analysis, coded allele frequency in entire dataset, beta value, meta-analysis OR (odds ratio), 1000 genomes minor allele frequency in Africans, Amerindians, Asians, and Europeans, evidence of DNA hypersensitive site (from Haploreg), Regulome DB score, GWAS hits from other diseases, ENCODE-described motifs which interact at the site, RefSeq gene annotation at that location, and the Location within the gene.

*Regulome DB Score

1a	eQTL + TF binding + matched TF motif + matched DNase Footprint + DNase peak
1b	eQTL + TF binding + any motif + DNase Footprint + DNase peak
1c	eQTL + TF binding + matched TF motif + DNase peak
1d	eQTL + TF binding + any motif + DNase peak
1e	eQTL + TF binding + matched TF motif
1f	eQTL + TF binding / DNase peak
2a	TF binding + matched TF motif + matched DNase Footprint + DNase peak
2b	TF binding + any motif + DNase Footprint + DNase peak
2c	TF binding + matched TF motif + DNase peak
3a	TF binding + any motif + DNase peak
3b	TF binding + matched TF motif
4	TF binding + DNase peak
5	TF binding or DNase peak
6	other (minimal binding data)
7	no data

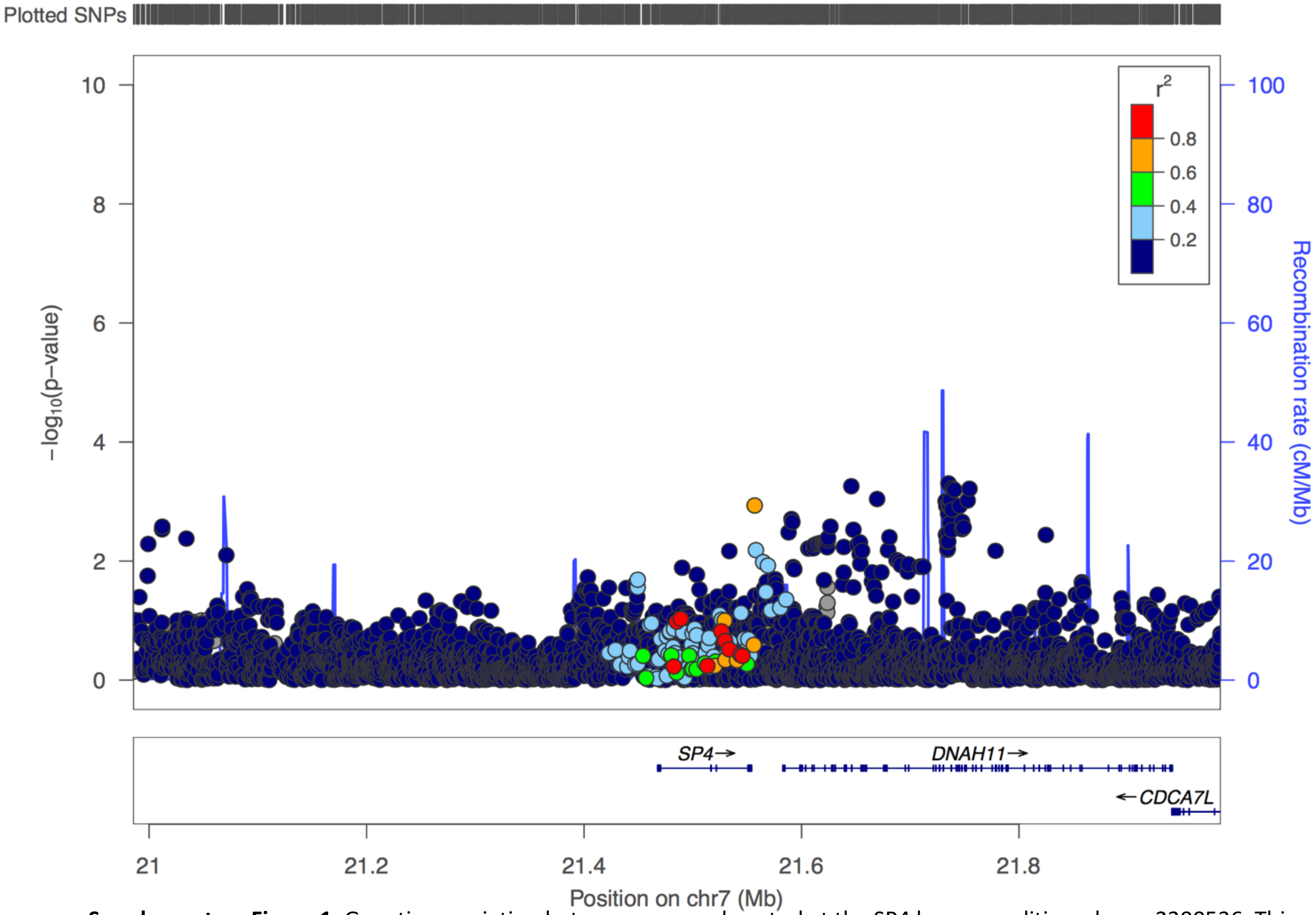

Supplementary Figure 1: Genetic association between case and control at the SP4 locus, conditioned on rs2390536. This represents the same region as displayed in Figure 2A of the main text.

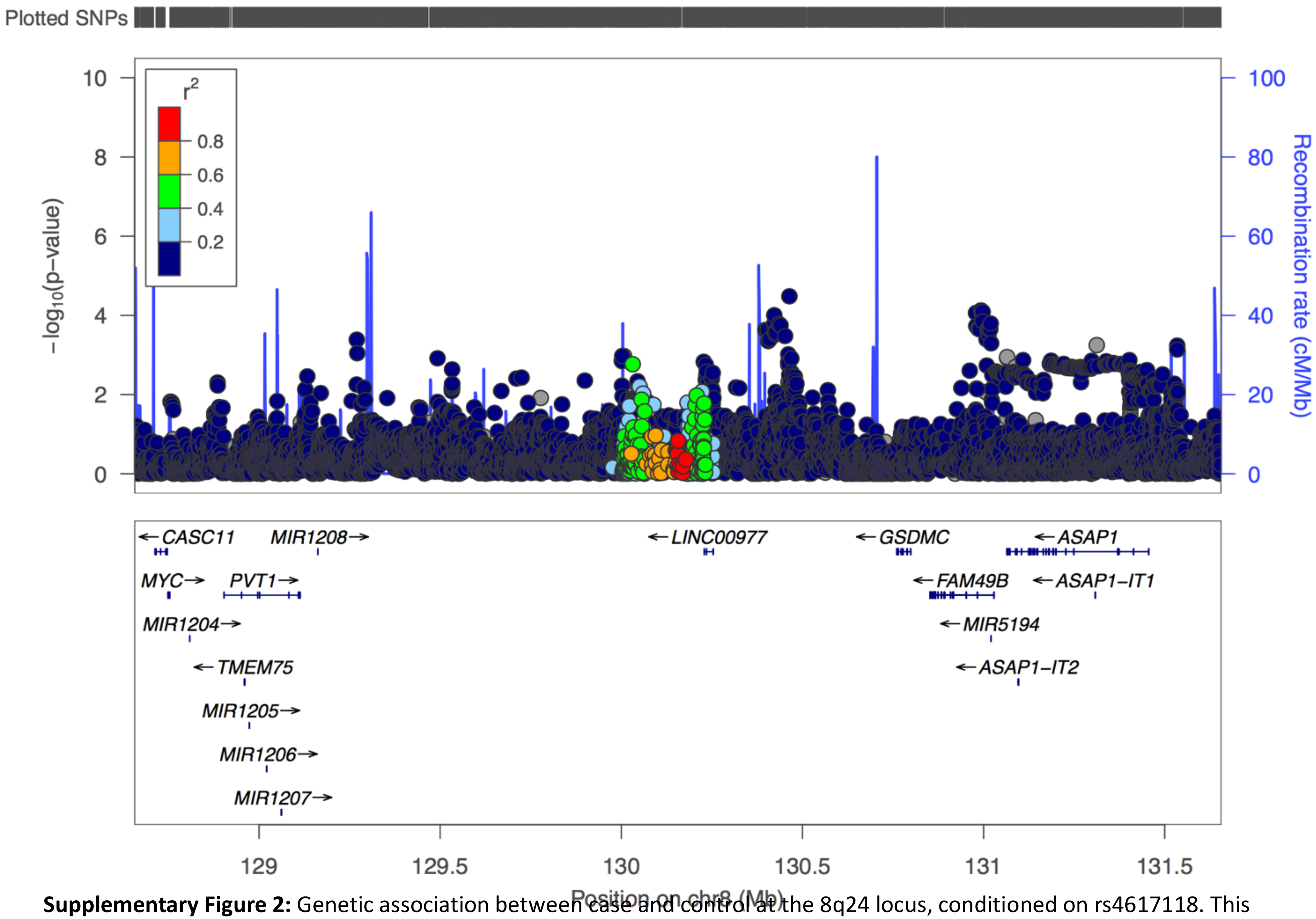

Supplementary Figure 2: Genetic association between case and control at the 8q24 locus, conditioned on rs4617118. This represents the same region as displayed in Figure 2B of the main text.

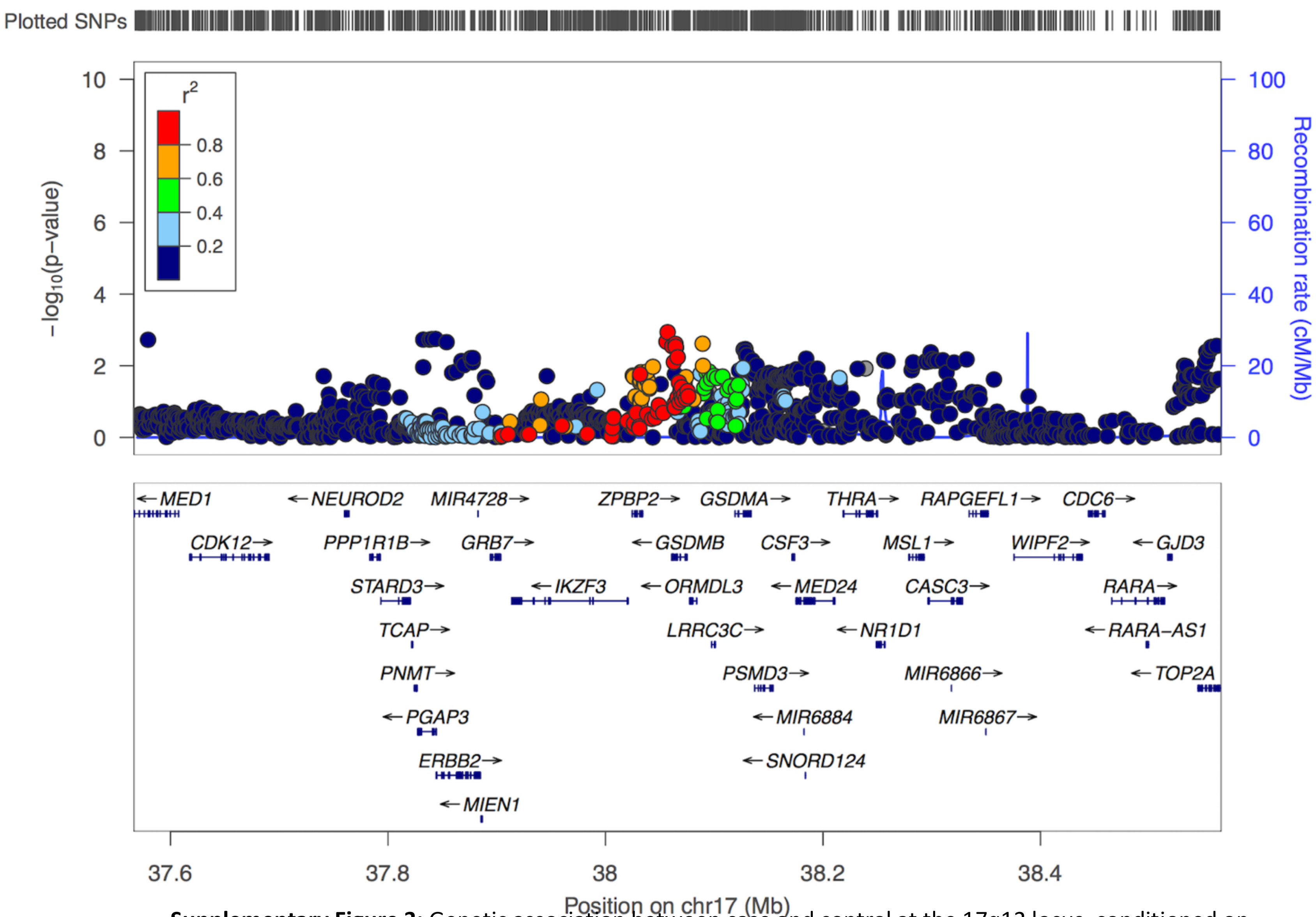

Supplementary Figure 3: Genetic association between case and control at the 17q12 locus, conditioned on rs2290400. This represents the same region as displayed in Figure 2C of the main text.
